# Spontaneous Terminal Ileum GIST Perforation Causing an Acute Abdomen in an Elderly Patient—A Rare Case

**DOI:** 10.3390/diagnostics15141816

**Published:** 2025-07-18

**Authors:** Marko Zivanovic, Milica Mitrovic-Jovanovic, Katarina Stosic, Nemanja Bidzic, Dragan Vasin, Danijela Sekulic, Jovan Peric, Milan Zuvela, Teodor Vasic, Danijel Galun

**Affiliations:** 1 Department for HBP Surgery, Clinic for Digestive Surgery, University Clinical Centre of Serbia, Koste Todorovica Street, No. 6, 11000 Belgrade, Serbia; nemanjabidzic@yahoo.com (N.B.); milan.zuvela011@gmail.com (M.Z.); teodorvasic@icloud.com (T.V.); galun95@gmail.com (D.G.); 2 Center for Radiology and Magnetic Resonance Imaging, University Clinical Centre of Serbia, Pasterova No. 2, 11000 Belgrade, Serbia; dr_milica@yahoo.com (M.M.-J.); katestosic@gmail.com (K.S.); draganvasin@gmail.com (D.V.); dacasekulic@gmail.com (D.S.); 3 Faculty of Medicine, University of Belgrade, Dr Subotica No. 8, 11000 Belgrade, Serbia; 4 Center for Anesthesiology and Resuscitation, University Clinical Center of Serbia, 11000 Belgrade, Serbia; jovan.peric994@gmail.com

**Keywords:** gastrointestinal stromal tumor (GIST), spontaneous perforation, tumor rupture

## Abstract

Gastrointestinal stromal tumors (GISTs) are rare mesenchymal tumors primarily located in the stomach and small intestine; their occurrence in the terminal ileum is particularly rare. Although GISTs can develop throughout the gastrointestinal tract, cases of perforation in elderly individuals are even less common, posing significant diagnostic and therapeutic challenges. This case report describes an 86-year-old male patient with an acute abdomen caused by a terminal ileum perforated GIST requiring urgent surgical intervention. An immunohistochemical examination of the tumor confirmed a GIST with a GILT (gastrointestinal leiomyogenic tumor) immunophenotype. The rarity of this condition makes it diagnostically challenging, as its symptoms are often nonspecific, and GISTs are frequently overlooked, particularly in older patients. This case supplements the existing literature by emphasizing the importance of considering GIST perforation in the differential diagnosis of an acute abdomen, even in elderly patients and in rare anatomical locations.

**Figure 1:**
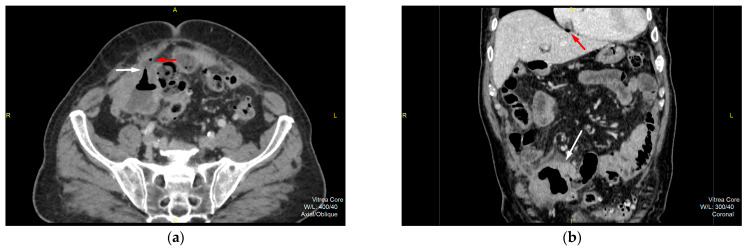
An 86-year-old male patient was admitted to the emergency hospital unit presenting with severe, diffuse, stabbing abdominal pain that started several hours prior to admission. His medical history included a previous appendectomy and cholecystectomy for inflammatory benign diseases. The physical examination demonstrated a tender abdomen with rebound tenderness and rigidity, predominantly in the lower abdominal region. This was accompanied by a high fever (up to 39 °C) and a pulse rate of 103 bpm. The laboratory tests showed elevated inflammatory markers, including a total WBC count of 13.2 × 10^9^/L with neutrophil predominance, an elevated CRP level of 167.1 mg/L, and a procalcitonin level of 2.170 ng/L, indicating a high risk of systemic infection. Abdominal ultrasound showed a large, heteroechoic, irregular formation in the right iliac region, which was closely associated with surrounding intestinal structures and laminar free fluid. Subsequently, the patient was referred for a multidetector computerized tomography (MDCT) scan. The axial MDCT scan (**a**) shows clearly demarcated thickening of the wall of the terminal ileum near the ileocecal valve with an irregular lumen and aeroliquid content. The tumorous segment of the terminal ileum loop shows a uniform tissue density with a hyperechoic surrounding fat plane, due to edema. Anteriorly, a gas-filled tubular tract (white arrow) is presented, which corresponds to the ileal wall rupture site with extraluminal free gas (red arrow). The coronal reconstruction (**b**) demonstrates wall irregularity with extensive reaction of the surrounding adipose tissue (white arrow) complicated by perforation due to a direct sign of a pneumoperitoneum—subphrenic free gas particles (red arrow).

**Figure 2:**
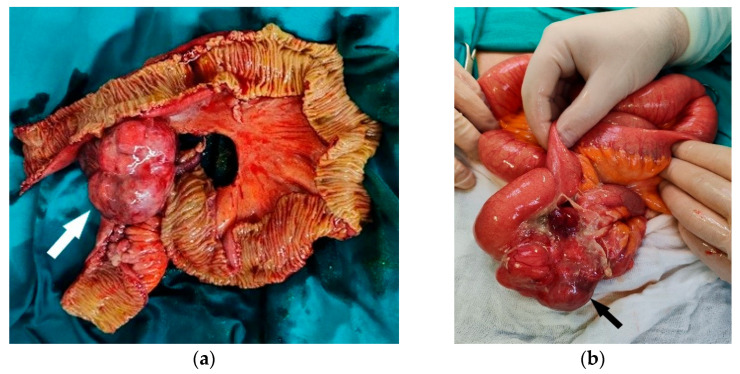
The MDCT features of a GIST are primarily a clear outer contour of the tumor, submucosal presentation, and relative tissue homogeneity in smaller tumors. With an increase in diameter, the structure of the tumor stroma degrades towards cystic and necrotic, which damages the intestinal or stomach mucosa itself and can lead to bleeding. Moreover, the risk of tumor rupture is increased, which leads to a worse prognosis of the disease and the spread of malignancy [1]. The main prognostic factors for disease recurrence based on several classification systems are the following: the tumor diameter, the mitotic index, tumor localization, and the disease stage [1,2,3,4]. The modified National Institutes of Health consensus criteria add tumor rupture as a new factor that classifies the tumor as a high-risk GIST [4]. Detection of the tumor rupture is of great importance for planning surgical intervention and for further oncological treatment [1]. The intraoperative findings showed a tumor-like alteration of the terminal ileum involving several loops of the small intestine. A resection of approximately 60 cm of the small intestine, including the corresponding mesentery, was performed, followed by stapled entero-enteric anastomosis. The postoperative course was uneventful with no signs of infection or anastomotic leakage. Due to the patient’s advanced age and comorbidities, no adjuvant therapy was initiated following a multidisciplinary oncological evaluation, particularly as the tumor was completely resected with negative margins. Radiological follow-up evaluations showed no evidence of disease dissemination or recurrence during the monitored period. (**a**) The resected specimen with opened small bowel loops revealing the tumor and the site of perforation. (**b**) An intraoperative view showing the tumor in situ at the moment of discovery.

**Figure 3:**
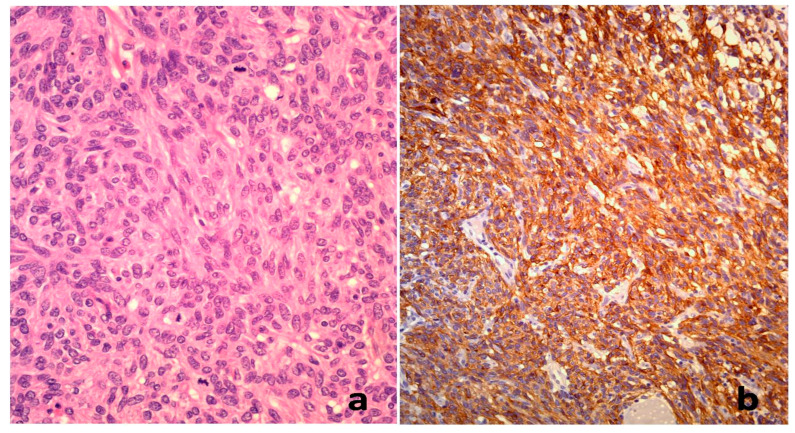
A pathohistological examination confirmed the diagnosis of a terminal ileum gastrointestinal stromal tumor (GIST). These findings correspond to the atypical immunophenotype noted in this rare case.Histopathological and immunohistochemical features of a gastrointestinal stromal tumor (GIST) in the terminal ileum, consistent with a GILT (gastrointestinal leiomyogenic tumor) immunophenotype. (**a**) Hematoxylin and eosin (H&E) staining at ×400 magnification showing spindle-shaped tumor cells with mild nuclear atypia, arranged in short fascicles. (**b**) Immunohistochemical staining at ×400 magnification demonstrating positive expression of smooth muscle actin and a voltage-gated calcium-activated chloride and bicarbonate channel, with only slight reactivity for CD117 (c-Kit) and absence of CD34 expression. The available publications confirm the correlation between the morphological MDCT features of GISTs and the high metastatic risk of these tumors [5,6]. The tumor diameter, the presence of interrupted mucosa, the tumor localization, a degraded tissue structure, irregular margins, and the tumor shape, as well as enlarged peritumoral vascular structures that feed and drain the tumor, are significant predictive MDCT factors of high-risk GISTs [7]. According to the current guidelines from the European Society for Medical Oncology (ESMO) and the National Comprehensive Cancer Network (NCCN), tumor rupture is considered a risk factor for disease recurrence and is associated with a dismal prognosis. In the presented case, the perforation of a terminal ileal GIST indicates a patient at high risk for peritoneal recurrence. Based on the current guidelines, the optimal management combines complete (R0) surgical resection followed by at least a three-year treatment with the adjuvant imatinib [8,9,10,11]. Several case reports have described the spontaneous perforation of gastrointestinal stromal tumors, but the majority of them have involved gastric or jejunal localizations [12,13]. Additional reports emphasized that perforated GIST of the terminal ileum can clinically mimic other intra-abdominal emergencies, such as inflammatory masses or septic peritonitis, particularly in elderly patients. The clinical presentation may include signs of diffuse peritonitis and hemodynamic instability that complicate preoperative diagnosis and delay definitive management. These findings further underline the importance of maintaining a high index of suspicion and acting promptly when imaging reveals features suggestive of a perforated neoplastic lesion in the lower abdomen [14,15]. In contrast, our case highlights a terminal ileum GIST, which is rarely the site of spontaneous perforation, especially in elderly patients. It is certainly a condition that requires urgent surgical intervention because it leads to acute abdominal symptoms, a risk of bleeding, and extensive peritonitis and sepsis [16]. The type of tumor and the signs of tumor perforation were recognized by means of a timely MDCT diagnosis, which further facilitated the surgical and oncological treatment of the patient [17]. Consequently, our patient—aged 86, with a spontaneously perforated GIST located in the terminal ileum and demonstrating an atypical immunophenotype—represents the oldest documented case of this specific presentation, further underscoring its novelty and clinical significance. The timely recognition of gastrointestinal stromal tumor (GIST) perforation based on characteristic MDCT findings is crucial for prompt surgical intervention and has a significant impact on patient outcomes.

## Data Availability

The datasets used and analyzed in this paper are available from the corresponding author upon reasonable request.
